# Call for a Mass Convention

**Published:** 1880-07

**Authors:** F. A. Levy

**Affiliations:** General Secretary


*Call for a Mass Convention of the Dentists of the United States to Organize a National Dental Association.*

At their annual meetings in 1879 the Southern Dental Association and the American Dental Convention each appointed a committee invested with full power to adopt measures for the formation of a National Dental Association. The American Dental Association at its meeting in the same year appointed a committee to confer with these two committees on this movement and report to the Association at its meeting in Boston, August 3, 1880. Members of these committees met at Saratoga in August, 1879, and elected Professor J. Taft, General Chairman.

The committees are as follows :

SOUTHERN DENTAL ASSOCIATION.

| | |
|---|---|
| R. Finley Hunt, Chairman, | Washington, D. C. |
| T. T. Moore, | Columbia, S. C. |
| S. J. Cobb, | Nashville, Tenn. |
| T. S. Waters, | Baltimore, Md. |
| J. R. Walker, | New Orleans, La. |
| F. J. S. Gorgas, | Baltimore, Md. |

AMERICAN DENTAL CONVENTION.

| | |
|---|---|
| R. B. Winder, Chairman, | Baltimore, Md. |
| J. G. Ambler, | New York, N. Y. |
| F. A. Levy, | Orange, N. J. |
| J. Taft, | Cincinnati, O. |

| | |
|---|---|
| F. Y. Clark, | Saratoga, N. Y. |
| C. S. Hurlbert, | Springfield, Mass. |

AMERICAN DENTAL ASSOCIATION.

| | |
|---|---|
| A. W. Harlan, Chairman, | Chicago, Ill. |
| C. N. Peirce, | Philadelphia, Pa. |
| F. H. Rehwinkel, | Chillicothe, O. |
| H. J. McKellops, | St. Louis, Mo. |

A meeting of Chairmen was held in New York, May 11, 1880, at which were present Professor R. B. Winder, Dr. R. Finley Hunt, and Professor C. N. Pierce, the latter representing (by proxy) Professor J. Taft, General Chairman. Professor Pierce was also present as a member of the commitee of the American Dental Association.

This meeting, in accordance with the powers and duties intrusted to the committees, decided to call a Mass Convention, and fixed the time and place of meeting, Wednesday, August 11, 1880, at 11 o'clock A. M., in the city of New York, for the purpose of organizing a National Dental Association, to be composed of members of every State society in the country.

A constitution and all necessary regulations will be prepared by a sub-committee, and submitted for revision to a meeting of the full committees, to be held in the same city at 9 o'clock A. M., on Monday, August 9, 1880, at Sturtevant House. so as to be thoroughly prepared for the action of the Mass Convention.

The importance of this measure requires that the whole time of the Convention shall be devoted to the business of organization.

All members of State societies and associations, and those intending to become members, are cordially invited to be present at this Mass Convention and take part in its proceedings.

As this proposed National Association is intended to promote the best interests of the whole profession of the country, it is important and is especially urged that every State in the Union be represented by as large a number of Dentists as possible.

It is hoped, therefore, that *every* dentist in the country who can *possibly* go to New York at that time will be present.

The following Executive Committees have been appointed to act together and make all arrangements for this meeting, such as procuring a hall, hotel accomodations reduction of railroad fares, etc:

BY AMERICAN DENTAL CONVENTION.

| | |
|---|---|
| J. G. Ambler, | New York, N. Y. |
| Charles Merritt, | " " " |
| H. Townsend, | Philadelphia, Pa. |
| J. H. Smith, | New Haven, Conn. |
| E. D. Fuller, | Peekshill, N. Y. |
| G. A. Mills, | Brooklyn, N. Y. |

BY SOUTHERN DENTAL ASSOCIATION.

| | |
|---|---|
| W. H. Atkinson, | New York, N. Y. |
| S. J. Cobb, | Nashville. Tenn. |
| J. W. Selby, | New York, N. Y. |

BY NEW YORK STATE DENTAL SOCIETY, to co-operate with other committees.

| | |
|---|---|
| O. E. Hill, | Brooklyn, N. Y. |
| L. S. Straw, | Newburgh, N. Y. |

J. TAFT, General Chairman.
R. B. WINDER, Chairman Committee of A. D. C.
R. FINLEY HUNT, Chairman Com. So. Dental Ass'n.
F. A. Levy, General Secretary.

NATIONAL DENTAL ASSOCIATION.

The Executive Committee of the National (late Southern) Dental Association, of the American Dental Association, and of the New York State Society, would respectfully announce to the dentists of the United States that they have made ample provision for the reception and entertainment of all dentists, whether alone or with their families, who will visit New York at the time of the assembling of the Convention, from August 9th to 14th. The following hotels ars recommended as being favorably situated for the convenience of dentists: Sturtevant House, Broadway, near 29th street; Coleman House, Broadway, near 28th street; Continental Hotel, corner Broadway and 20th street; Rossmore Hotel, corner Broadway and 42d street; Grand Central Hotel, Broadway and 3d street. The above hotels will accommodate guests with rooms at the rate of $1 per night for each person, and $2.50 for room and board for each person. Parlors have been offered at Sturtevant House, Coleman House and the Continental for the use of the committees. On and after the morning of August 9th, a committee will be in attendance at Sturtevant House for the purpose of giving visitors information on all points connected with the Convention.

EXCURSION FARES.—The railroad excursion arrangements by the lines from the East, West and Northwest are so ample and the rates so low, that it was not necessary to make special arrangements.

From Southern points the excursion rates to New York and return will be as follows: Norfolk, Va., by steamer, $14; Charleston, S. C., by steamer, $25: Augusta, Ga., via Charleston steamer, $30; Atlanta, Ga., via Charleston steamer, $40.35; Macon, Ga., at the rate of 6c. per mile one way to Augusta, thence by Charleston. Other railroad rates not yet arranged for will be reported through the Presidents of the several state societies.

There will several excursions given by the dentists of New York and vicinity, in which all visiting dentists are invited to participate, with their families, which will without doubt add to the pleasure of the meeting.

(Signed) F. A. LEVY,
General Secretary.

